# Seasonal Sexual Segregation by Monomorphic Sooty Shearwaters *Puffinus griseus* Reflects Different Reproductive Roles during the Pre-Laying Period

**DOI:** 10.1371/journal.pone.0085572

**Published:** 2014-01-09

**Authors:** April Hedd, William A. Montevecchi, Richard A. Phillips, David A. Fifield

**Affiliations:** 1 Cognitive and Behavioural Ecology Program, Psychology Department, Memorial University, St. John’s, Newfoundland and Labrador, Canada; 2 British Antarctic Survey, Natural Environment Research Council, Cambridge, United Kingdom; Hawaii Pacific University, United States of America

## Abstract

Tracking technology has revolutionized knowledge of seabird movements; yet, few studies have examined sex differences in distribution and behavior of small to medium-sized, sexually-monomorphic seabirds. Application of bird-borne geolocation-immersion loggers revealed seasonal segregation in the sexually-monomorphic Sooty Shearwater *Puffinus griseus*, mainly in the pre-laying period, when there were clear differences in reproductive roles. Shearwaters first returned to the Falkland Islands on 27 Sept±8 d; males, on average, 8 d earlier than females. Prior to egg-laying, distribution at sea, colony attendance and behaviour depended on sex. Males foraged locally over the southern Patagonian Shelf and Burdwood Bank, spending mainly single days at sea and intervening nights in the burrow. Females, who flew for more of the day during this time, foraged in more distant areas of the northern Patagonian Shelf and Argentine Basin that were deeper, warmer and relatively more productive. Attendance of females at the colony was also more variable than that of males and, overall, males were present for significantly more of the pre-laying period (38 vs. 19% of time). Sex differences were reduced following egg-laying, with males and females using similar foraging areas and making trips of similar mean duration in incubation (7.6±2.7 d) and chick-rearing (1.4±1.3 d). Congruence continued into the non-breeding period, with both sexes showing similar patterns of activity and areas of occupancy in the NW Atlantic. Thus, seasonal changes in reproductive roles influenced patterns of sexual segregation; this occurred only early in the season, when male Sooty Shearwaters foraged locally, returning regularly to the colony to defend (or maintain) the burrow or the mate, while females concentrated on building resources for egg development in distant and relatively more productive waters.

## Introduction

Development of miniaturized tracking devices have been used to document sex differences in distribution and behavior of large seabirds with pronounced sexual size-dimorphism, such as Wandering Albatrosses *Diomedea exulans* [1]–[3] and Giant Petrels *Macronectes* spp. [4], [5]. There have, however, been very few studies of sex differences in small to medium-sized monomorphic species, including the Procellariiformes. Reviewing patterns of seabird sexual segregation in both distribution at sea (inferred from δ^13^C) and trophic position (δ^15^N), Phillips et al. [6] reported an apparent link between sexual size dimorphism and segregation: segregation was relatively common in dimorphic species and extremely rare in species where males and females are similar in size. Sex differences were also more common during pre-laying or breeding than they were during the non-breeding period, likely reflecting a greater need for resource partitioning when birds are subject to central place foraging constraints. Phillips et al. [6] also highlighted mechanisms likely resulting in sexual segregation, including size-mediated competitive exclusion, habitat or dietary specialization.

The decreasing size and increasing recording capacity of geolocation and other devices has produced a surge of information on movements and behaviour for a diversity of species during migration, opening a window into the least known period of the annual cycle [7]–[15]. Particularly for small to medium-sized species, this research has greatly widened our perspective on sexual segregation, including an increasing number of reports for more monomorphic species [14], [16]–[20]. Explanations for sex-specific foraging in the absence of sexual size dimorphism have included inter-sexual competition potentially arising from sex differences in foraging efficiency [16], reproductive role specialization whereby the relative parental contributions of males and females differ [20], and mechanisms whereby differential parental investment results in sex-specific energetic or nutritional requirements during particular breeding phases [14], [17], [18]. The degree of sexual segregation in most monomorphic species and our understanding of the underlying or evolutionary mechanisms, however, remain largely unknown.

Here we examine sex differences in distribution, oceanographic characteristics and behavior of the sexually monomorphic Sooty Shearwater *Puffinus griseus* throughout its annual cycle. Geolocation-immersion loggers were used to record distributions at sea, along with foraging and behavioural parameters (colony attendance, trip durations, and activity patterns). Our objectives were to describe movement strategies and behavior of males and females across successive phases of the breeding and non-breeding season first to determine whether sexual segregation occurs in this species, and if so, whether any sex differences are constant through time.

## Materials and Methods

### Ethics Statement

Approval of the protocols used in this study was granted by Memorial University of Newfoundland’s Institutional Animal Care Committee (07-06-WM, 08-01-WM, 10-01-WM), and both Research Licenses (R21/2007, R11/2008) and Visitor Access permits for field work were granted by the Falkland Islands Government. The necessary permits were obtained and all regulations were followed.

### Logger Deployment

**Figure 1:**
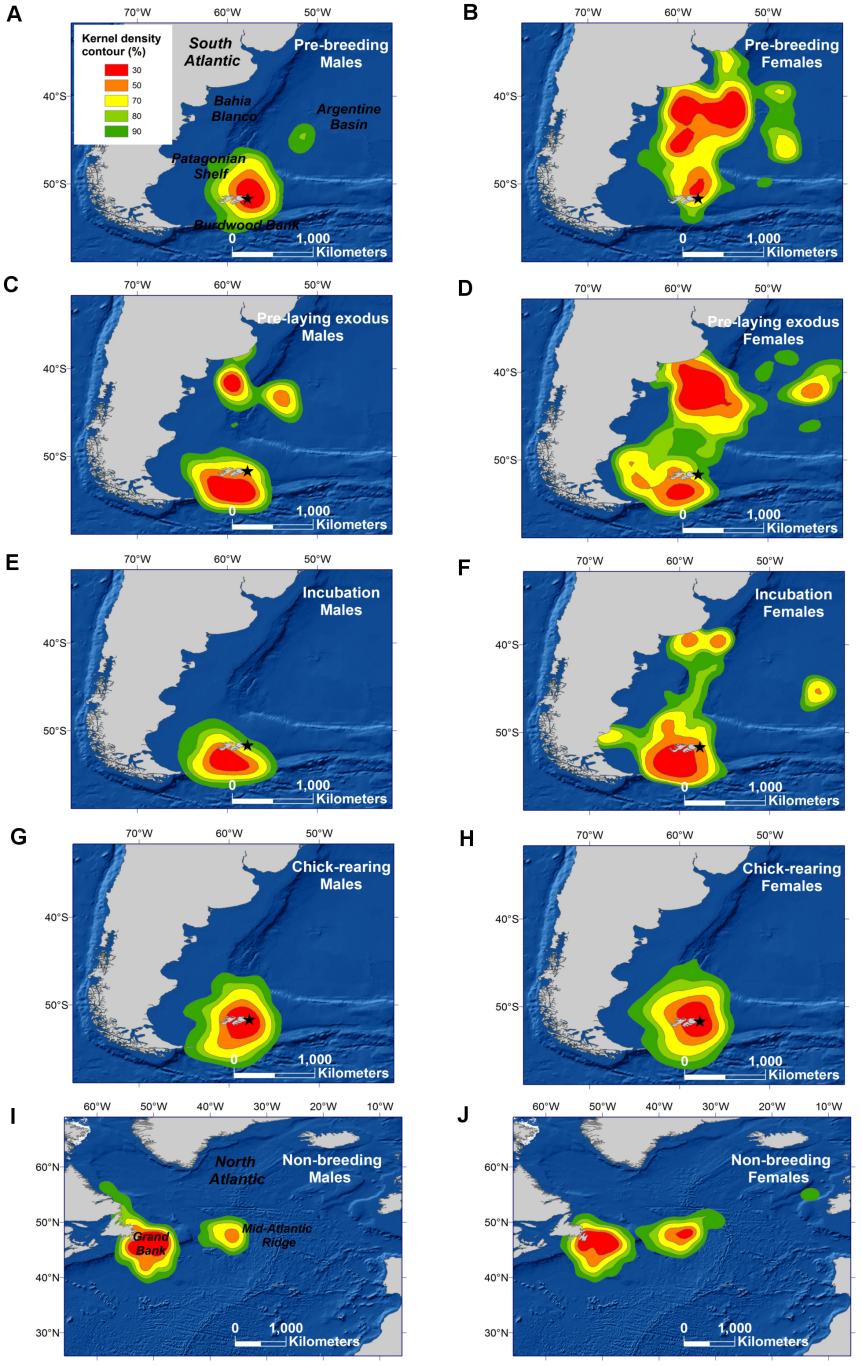
Spatial distribution of Sooty Shearwaters throughout the year. Kernel density distribution of male and female Sooty Shearwaters from Kidney Island, Falkland Islands (star) during the pre-breeding period (i.e., prior to the pre-laying exodus; A,B), the pre-laying exodus (C, D), the incubation (E, F) and chick-rearing periods (G, H), and the period of non-breeding residence (I, J). Place names mentioned in the text are indicated.

This study was conducted during the austral summers of 2007/08, 2008/09 and 2009/10 (hereafter the 2008, 2009 and 2010 seasons) in the Falkland Islands at Kidney Island (51° 37′ S, 57° 45′ W; Fig. 1), where >100,000 pairs of Sooty Shearwaters breed [21]. From 10–13 December 2007, we deployed geolocation-immersion (GLS) loggers attached to oval-shaped plastic bands on the tarsi of 44 adult shearwaters (each from a different burrow) during incubation. Individual breeding status was determined either from the presence of an egg at deployment or subsequently from patterns of colony attendance derived from the light and activity data. Device attachment, weighing (using a 1 kg Pesola spring balance) and banding (Canadian Wildlife Service stainless steel band) took ∼5 min, after which birds were returned to burrows. Study burrows were marked at the entrance with a metal tag and flagging tape, and left undisturbed for 12 months. Three types of GLS were used: British Antarctic Survey (BAS) Mk5 (logger+attachment = 5.1 g, ∼0.6% body mass, n = 15) which recorded light, temperature and immersion, BAS Mk13 (logger+attachment = 2.9 g, ∼0.3% body mass, n = 15) which recorded light and immersion only, and Lotek LAT 2500 archival loggers (logger+attachment = 5.9 g, ∼0.7% body mass, n = 14) which recorded light, temperature, and wet/dry state (the latter 2 parameters every 240 s throughout deployment). Burrows were visited during incubation and early chick-rearing in 2009 and 2010 to retrieve devices. Birds were weighed at recapture and ∼0.5 ml of blood was drawn from the brachial vein to determine sex using molecular techniques [22].

### Logger Processing and Analysis

As all datasets presented here were derived from BAS GLS loggers, the details of processing and analysis refer specifically to these devices. Light data were processed with TransEdit software (BAS, Cambridge, UK) using a light threshold of 10 and an angle of elevation of –4.7 [23]. Day/night length is used to provide an estimate of latitude, and Julian date plus the time of local midday/midnight relative to GMT is used to estimate longitude. This procedure produces two locations per day, corresponding to local midday and midnight. Mean positional error ± SD of similar devices deployed on free-ranging albatrosses was 186±114 km and 202±171 km [23], [24]. Latitude cannot be accurately estimated from light levels during equinox [25] (as daylength is approximately similar around the globe), though when possible latitude estimates were obtained by reconciling temperature data recorded by the loggers (BAS Mk5 only) with satellite remotely sensed sea surface temperatures (SSTs) [24], [26]. BAS GLS loggers record temperature (accuracy ±0.5° above 0°C, and ±1.0° from 0° to –10°C) only after 20 min of continuous immersion, providing stable surface (or subsurface) temperatures suitable for comparison with satellite derived SST data. Light-based latitudes that were clearly affected by proximity to equinox for records without corresponding temperature information (BAS Mk13 and 1 MK5) were excluded. Longitude estimates are unaffected by equinox and were retained to ascertain east-west movements during these times. As well, clearly erroneous positions, derived from light curves that were associated with interference around sunrise and sunset, were removed.

Light and immersion records were examined simultaneously in MultiTrace Geolocation (Jensen Software Systems, Laboe, Germany) to distinguish time at sea from time in the burrow (spells in the burrow were dark and dry) and to help infer the duration of foraging trips/burrow shifts and the timing of breeding and migration [27]. For instance, we assumed egg-laying date to coincide with the female’s return to the burrow following the pre-laying exodus, and hatch date to coincide with the initial decrease of parental foraging trip/burrow shift durations to ∼1–2 d. Sooty Shearwaters generally spend longer periods in the burrow during incubation (100% of n = 33 shifts were 3–13 d) than during chick-rearing (99% of n = 502 shifts were <2 d). Unlike some petrels, which make brief visits to the colony to feed chicks [14], [28], at this site at least, Sooty Shearwaters typically spend the night ashore when they return to feed chicks. Birds arrive in the colony soon after dark and stay until shortly before daylight (i.e., loggers record a continuous dry period of several hours), a behavior that enabled us to estimate foraging trip durations during chick-rearing. Validated spatial data from extended foraging trips were smoothed twice, with locations added on the dates of colony departure and return; these were determined from direct examination of the light data [29]. During extended pre-laying and incubation foraging trips, approximate maximum ranges (furthest distance from the colony) and cumulative travel distances (both great-circle) were calculated. Characteristically brief (<2 d) foraging trips typical of chick-rearing, and to some extent the pre-breeding period, were too short to smooth. To facilitate data analysis, the annual cycle was partitioned into the following stages: 1) pre-breeding (colony return to pre-laying exodus), 2) pre-laying exodus, 3) incubation, 4) chick-rearing, 5) outward and return migration and 6) non-breeding residence (end of the outward to start of the return migration; see [27] for details).

BAS GLS tested for saltwater immersion every 3 sec, and recorded the number of positive tests every 10 min as a value between 0 (all dry) and 200 (all wet). The immersion data were categorized into day (civil sunrise to civil sunset; sun 6° below horizon) and night (civil sunset to civil sunrise) and used to determine the proportion of time birds spent flying and sitting on the sea. Time budget calculations excluded periods spent in the burrow (prolonged dark and dry periods). Each day, the 10 min periods spanning day to night and night to day were removed prior to analysis.

Locations of the birds at sea were mapped in ArcGIS 9.3 (ESRI, Redlands, CA, USA), and Spatial Analyst and Hawth’s Tools, respectively, were used to create kernel density surfaces (North or South Pole Lambert azimuthal equal-area projection; cell size = 50 km, search radius = 200 km) and percent volume contours to describe utilization distributions (UDs) [30]. Kernel density maps were produced for each stage defined above (except migration) with 50% UDs describing core or high use areas. Spatial overlap of core areas for males (*A_M_*) and females (*A_F_*) was determined by overlaying 50% UDs and calculating the area of overlap (*A_O_*, km^2^) using the Intersect tool in ArcMap 9.3 (ESRI, Redlands, CA, USA). The percent of core area shared by males and females (which could range from 0–100%) followed this equation [15]:
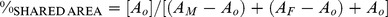



The influence of sex, stage, and their interaction, on foraging trip characteristics and immersion patterns of shearwaters were examined using general linear mixed effects models (LME) fit by restricted maximum likelihood. To improve normality of the immersion data, proportions were logit transformed prior to analysis [31]. Mixed modeling was employed to account for potential pseudoreplication, with individual set as a random effect. *F*-tests were used to assess the significance of effects. When assumptions of parametric tests were violated, nonparametric tests were used to assess significance of effects using median parameter values for individual birds. Models were built and statistics were run using R software [32], and unless stated otherwise, values are presented as means ±1 standard deviation.

### Oceanographic Characteristics

A broad description of the oceanography of areas occupied by Sooty Shearwaters was obtained by overlaying kernel UDs onto maps of bathymetry and remotely-sensed sea surface temperature (SST) and chlorophyll concentration (chl *a*). Bathymetry was determined using ETOPO2 grids (http://www.ngdc.noaa.gov/mgg/global/etopo2.html). SST and chl *a* were monthly composite Aqua MODIS mapped products at 9 km resolution, downloaded from http://oceancolor.gsfc.nasa.gov. Oceanographic data for the period prior to egg-laying (pre-breeding and pre-laying exodus) were from October and November 2008, respectively, and for the longer incubation, chick-rearing and non-breeding phases extractions were from representative middle months (December, February and July 2008, respectively).

## Results

### Data Availability

Our dataset includes information on colony attendance, distribution and immersion (i.e., wet-dry activity) for 17 shearwaters (10 females, 7 males; n = 10 BAS Mk5 and n = 7 BAS Mk13 records). To eliminate potentially confounding effects of inter-year variability, only data collected during the first year (incubation 2008 to egg-laying 2009) were included. There was no evidence of sexual size dimorphism in our sample of Sooty Shearwaters: males (897±87 g) and females (881±50 g) had similar mean mass at deployment (*F*_1,15_ = 0.24, *p* = 0.63).

### Colony Attendance and Distribution at Sea

As there was a significant sex by stage interaction for foraging trip duration (*F*_3,690_ = 55.50, *p*<.0001) and, in addition, the influence of sex on distribution depended on the time of year, results for each stage are presented separately.

#### Pre-breeding and pre-laying exodus

**Figure 2:**
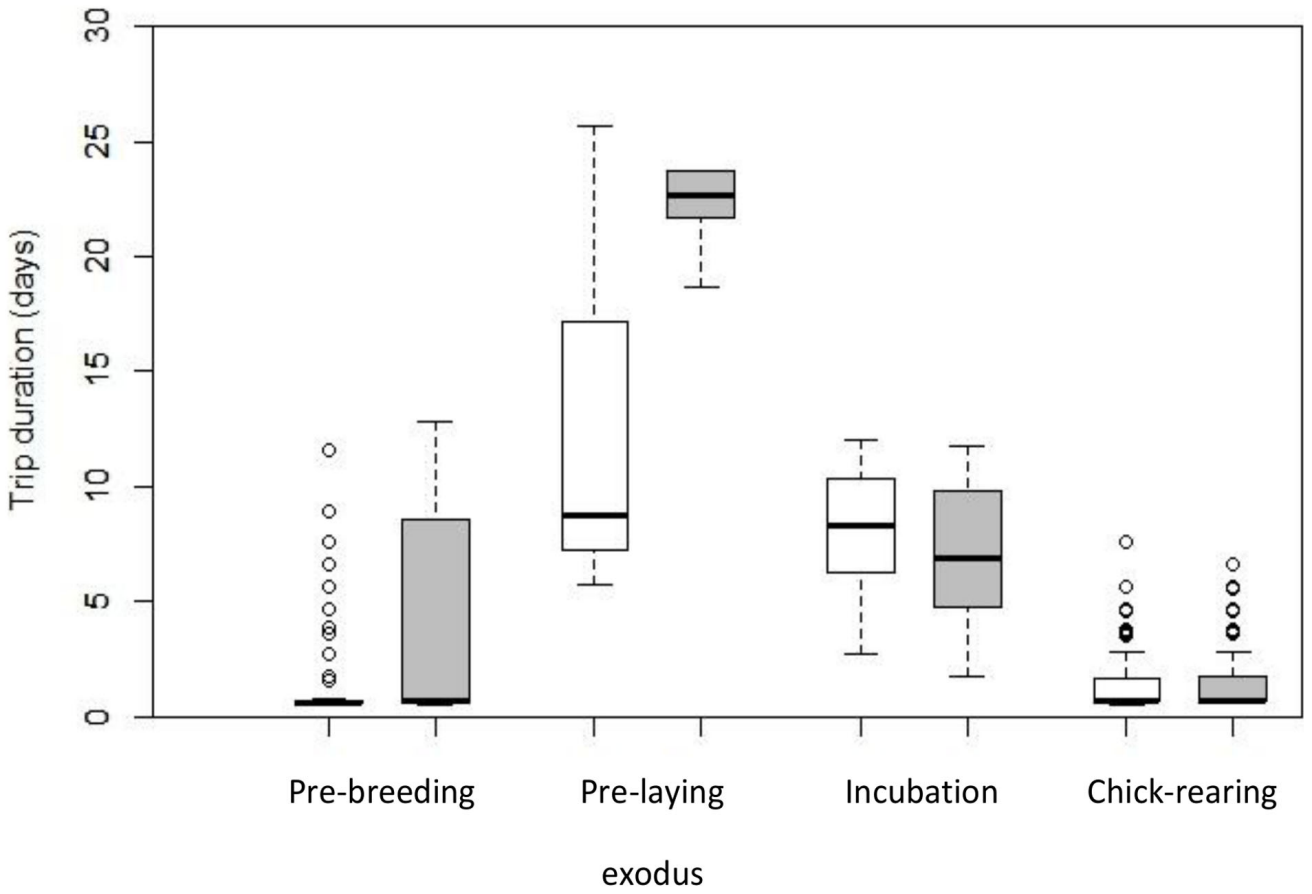
Foraging trip durations of Sooty Shearwaters during the pre-breeding and breeding periods. Males are depicted in white and females in grey. Box plots show the median and 25^th^ and 75^th^ percentiles, whiskers indicate values within 1.5 times the interquartile range and circles represent outliers.

Shearwaters first returned to the breeding colony on 27 September ±8 d (13 September –11 October, n = 16); males, on average, 8 d earlier than females (*F*_1,14_ = 6.01, *p* = 0.028). Although all birds regularly attended the colony during the pre-breeding period (the interval between first return and departure on the pre-laying exodus), attendance patterns varied by sex. Males adopted daily routines during this time, typically spending single days at sea and intervening nights in the burrow (Fig. 2). The duration of female foraging trips, in contrast, was more variable (Fig. 2), and overall, the distribution of trip durations differed between the sexes (Mann-Whitney *U* = 9.0, df = 1, *p* = 0.014).

All shearwaters performed a pre-laying exodus in 2009, leaving the colony on average on 02 November (±5 d, 24 October –08 November, n = 16) and returning 18±8.1 days later (20 November ±4 d, 12–24 November, n = 16; Table 1 and Fig. 2). Mean exodus duration was significantly longer for females (22.4±3.5 d) than males (12.0±8.7 d; *F*_1,14_ = 10.89, *p* = 0.005); females also ranged further from the colony (1,474±419 *vs* 707±466 km; Mann-Whitney *U* = 7.0, df = 1, *p* = 0.010) and travelled greater total distances (5,906±1,412 *vs* 2,830±1,997 km; Mann-Whitney *U* = 9.0, df = 1, *p* = 0.017). Between colony return and the end of the pre-laying exodus, males spent significantly more time attending the colony than females (38 [range 17–52] *vs* 19% [range 14–24] of total time, respectively; *F*_1,14_ = 20.55, *p*<0.001). Hence, females spent a greater proportion of their time at sea (81 [range 76–86] *vs* 62% [range 48–83] for males).

**Table 1:** Characteristics of the pre-laying exodus of Sooty Shearwaters tracked using GLS loggers from Kidney Island, Falkland Islands, prior to the 2009 breeding season.

Band	Sex	Colony	Exodus		Duration	Distance	Maximum	Main destination
		return date	Start date	End date	(d)	(km)	range (km)	
6073	F	11-Oct-08	31-Oct-08	22-Nov-08	22	6120	1620	N Patagonian Shelf
6092	M	30-Sept-08	25-Oct-08	18-Nov-08	24	6580	1120	Argentine Basin
6097	F	06-Oct-08	02-Nov-08	23-Nov-08	21	5620	1580	N Patagonian Shelf/Slope+Argentine Basin
6079	M	27-Sept-08	07-Nov-08	12-Nov-08	5	1660	520	S Patagonian Shelf
6095	F	29-Sept-08	30-Oct-08	22-Nov-08	23	7310	1790	N Patagonian Slope+Argentine Basin
6076	F	08-Oct-08	24-Oct-08	24-Nov-08	31	8000	1290	N Patagonian Shelf/Slope+Argentine Basin
6090	F	25-Sept-08	30-Oct-08	22-Nov-08	23	7250	1990	Argentine Basin (central)
6077	F	02-Oct-08	31-Oct-08	21-Nov-08	21	4480	1070	(N Patagonian Shelf)+Burdwood Bank
6093	M	24-Sept-08	04-Nov-08	13-Nov-08	9	2380	400	Burdwood Bank
6085	F	18-Sept-08	27-Oct-08	17-Nov-08	21	4260	650	S Patagonian Shelf
3622	F	01-Oct-08	03-Nov-08	21-Nov-08	18	4200	1450	N Patagonian Shelf/Slope
6100	M	16-Sept-08	08-Nov-08	16-Nov-08	8	1710	460	S Patagonian Shelf
6091	F	27-Sept-08	31-Oct-08	22-Nov-08	22	5910	1830	Argentine Basin (central)+(Burdwood Bank)
3623	M	23-Sept-08	25-Oct-08	19-Nov-08	25	4590	1590	N Patagonian Shelf
6083	M	13-Sept-08	08-Nov-08	13-Nov-08	5	1210	490	Burdwood Bank
6088	M	25-Sept-08	08-Nov-08	16-Nov-08	8	1680	370	Burdwood Bank
Mean		27-Sept	02-Nov	20-Nov	18	4,560	1,140	
SD		7.7	5.1	3.9	8.1	2,270	580	

**Figure 3:**
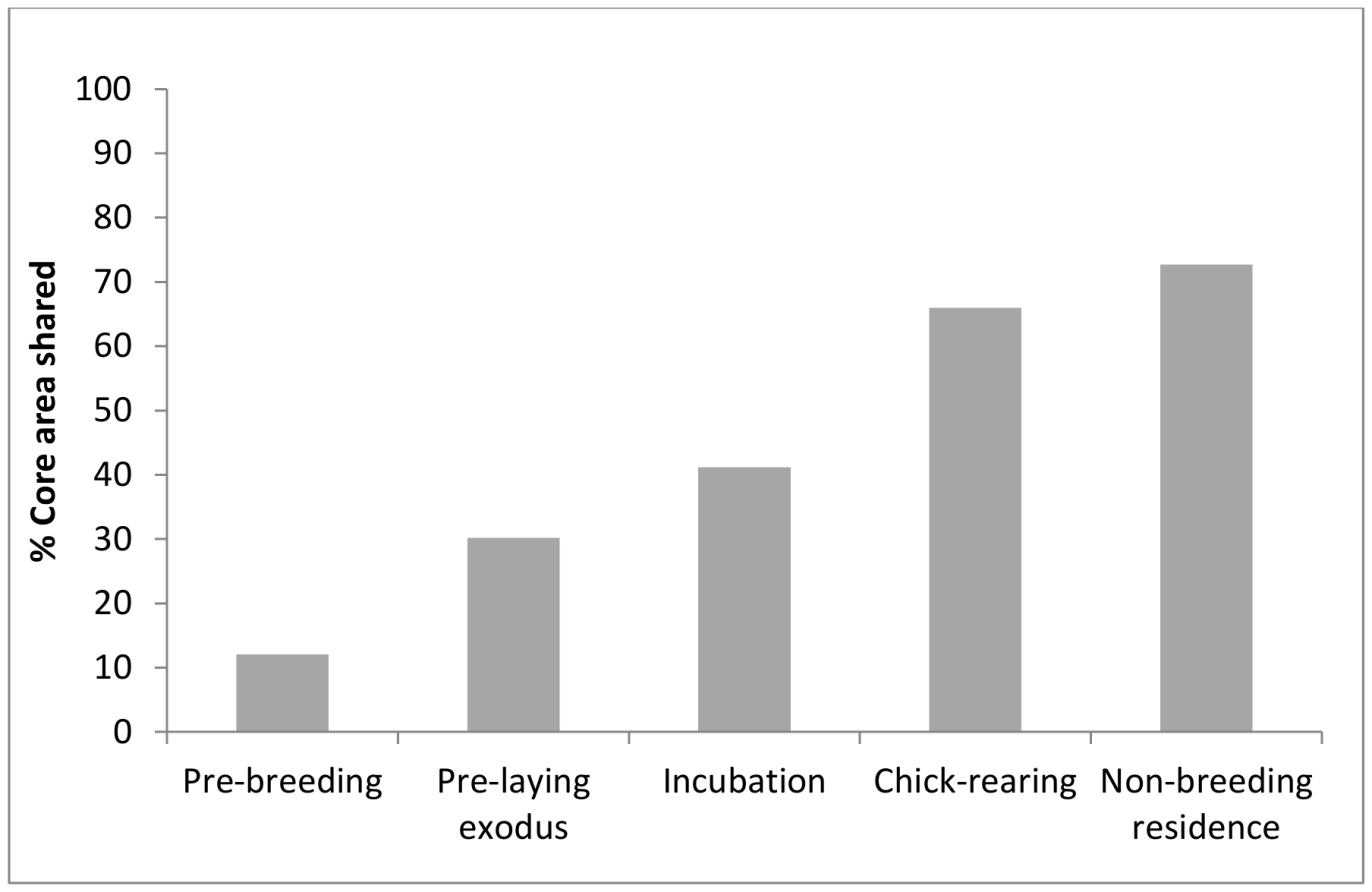
Percentage of core foraging habitat shared by male and female Sooty Shearwaters.

**Figure 4:**
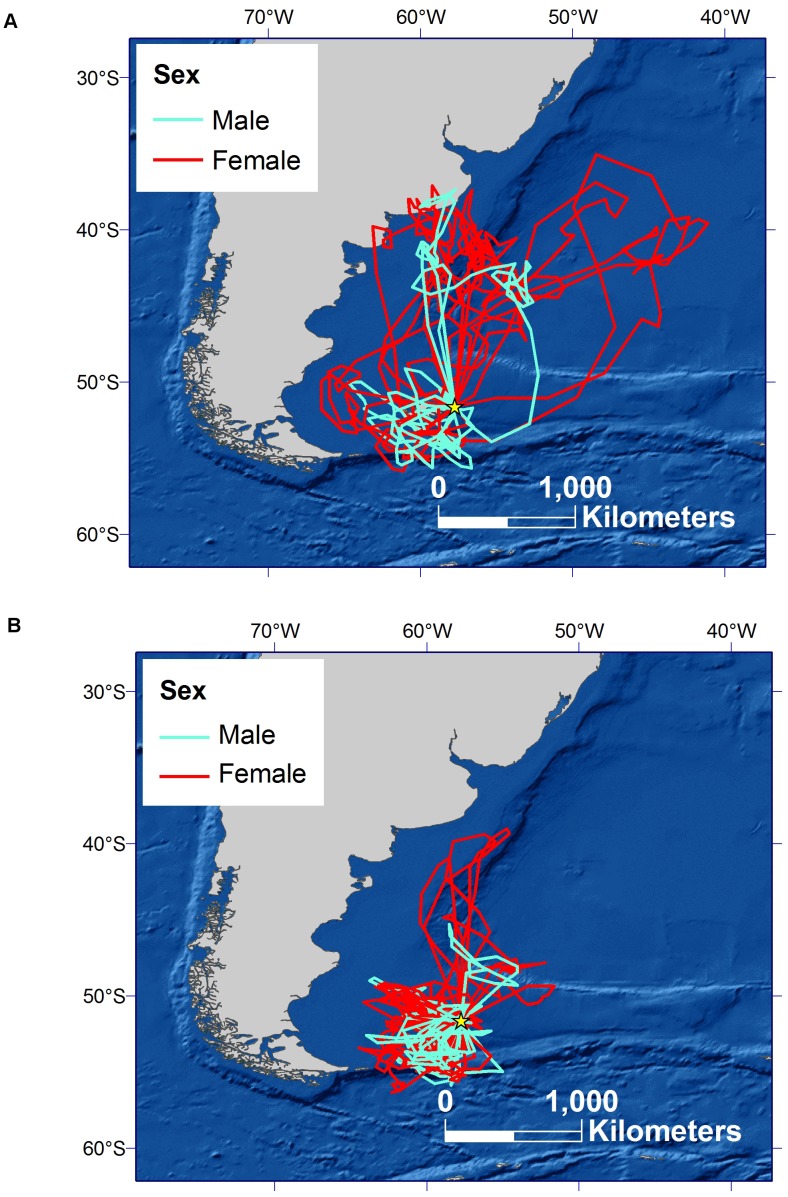
Foraging routes of individual Sooty Shearwaters during the pre-laying exodus (A) and incubation (B) periods.

Prior to egg-laying, core foraging areas (50% UDs) spanned the Burdwood Bank in the south to the northern Patagonian Shelf and slope, and adjacent pelagic waters of the Argentine Basin, east of Bahia Blanco (Fig. 1 A–D). Sex differences in colony attendance during this time were reflected in patterns of distribution at sea. Before (Fig. 1 A–B) and during the pre-laying exodus (Table 1 and Fig. 1 C–D), distant northern parts of the range were used heavily by females, and southern areas closer to the colony by males. Consequently, the percentage of core area shared by the sexes was low; 12% and 30%, respectively, prior to and during the pre-laying exodus (Fig. 3). Seven of 9 pre-laying exodus trips by females were focused over northern areas whereas on 5 of 7 trips males travelled to the southern Patagonian Shelf and Burdwood Bank (Fig. 4A).

#### Incubation

The mean estimated egg-laying date in 2009 was 22 November (±2 d, 17–24 November, n = 9). On average during incubation, foraging trips were 7.6±2.7 d (1.8–12.0 d, n_trips_ = 40, n_birds_ = 17; Fig. 2), to a mean maximum range of 491±273 km (109–1,403 km, n_trips_ = 39) from the colony and involved a total travel distance of 1,549±736 km (264–3,999 km, n_trips_ = 39; Fig. 4B). Trip durations, maximum foraging ranges and total distances covered during incubation were similar for males and females (*p*>0.33 for all LME model tests).

Relative to the pre-breeding period, core foraging areas during incubation were smaller and for both sexes they were centered over the southern Patagonian Shelf and Burdwood Bank, west and southwest of the colony (Fig. 1 E–F). Females continued to use the northern Patagonian Shelf, but less frequently and for shorter periods. As the ranges contracted, the percentage of the core area shared by the sexes increased to 41% (Fig. 3).

#### Chick-rearing

The average estimated hatching date in 2008 was 18 January (±6 d, 11 January –01 February, n = 15). Foraging trips during the chick-rearing period were short, averaging 1.4±1.3 d (0.5–10.6 d, n_trips_ = 496, n_birds_ = 14; Fig. 2) and of similar duration for both sexes (*F*_1,11_ = 0.13, *p* = 0.72). As GLS devices produce just 2 locations per day, trip brevity precluded spatial resolution at the individual trip level. Based on the pooled data, a single core area was apparent during chick-rearing, centered over the southern Patagonian Shelf (Fig. 1 G–H) at a maximum of ∼410 km from the colony. The degree of overlap between the sexes was high, at 66% (Fig. 3).

#### Non-breeding residence

Following rapid trans-equatorial migration, Sooty Shearwaters spent most of their non-breeding period in the Northern Hemisphere (mean residency 143 d [27]). Their distribution was initially centered to the west of the Mid-Atlantic Ridge in the region occupied at the end of the outward migration. Birds subsequently moved to the eastern Canadian Grand Bank and remained there throughout the northern summer (Fig. 1 I–J). The proportion of core habitat sharing by males and females reached a seasonal maximum of 73% (Fig. 3).

### At Sea Activity Patterns

**Figure 5:**
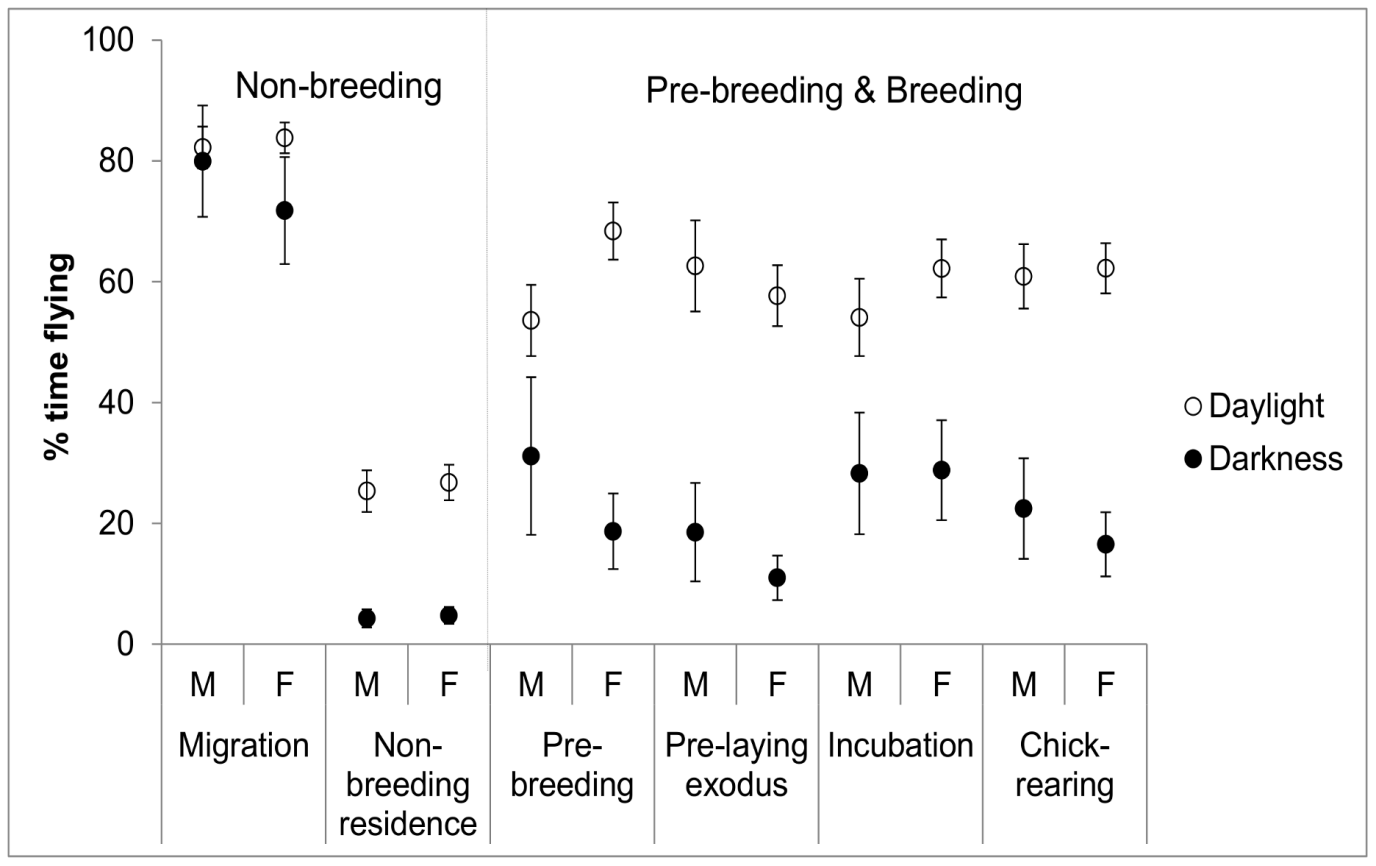
At sea activity patterns. Percent time spent flying by male and female Sooty Shearwaters during daylight and darkness in different stages of the annual cycle. Values are LME model fits ±95% confidence intervals.

Regardless of time of year, Sooty Shearwaters flew more during the day than at night (Fig. 5, Table 2). They flew most during migration (78% of the day and 59% of the night, on average), least when on the non-breeding grounds (29% and 11%, respectively), and at intermediate levels during the breeding season (57–60% and 22–33%, respectively; Table 2 and Fig. 5). Percentage of time spent flying during the day and during the night showed sex by stage interactions (*F*_5,4489_ = 5.93, *p*<0.0001 and *F*_5,3850_ = 3.49, *p*<0.004, respectively; Fig. 5). Females flew significantly more than males by day during the pre-breeding (*F*_1,13_ = 8.05, *p* = 0.014) and the incubation periods (*F*_1,13_ = 4.83, *p* = 0.047). For all other stages during the day, as well as for all stages at night, males and females had similar patterns of activity at sea.

**Table 2:** Activity patterns of Sooty Shearwaters, recorded by BAS GLS loggers, throughout the annual cycle.

	Pre-breeding	Pre-laying exodus	Incubation	Chick-rearing	Migration	Non-breeding residence
Time on water (%) during:						
Daylight	42.3±9.7	41.3±10.8	43.5±7.0	39.9±6.2	22.2±4.1	70.8±5.1
	(26.6–54.3)	(24.5–59.7)	(34.7–57.7)	(29.3–49.9)	(15.3–29.0)	(62.3–78.9)
Darkness	74.3±10.2	75.6±11.6	66.9±7.5	77.9±8.6	40.9±8.7	89.5±3.8
	(55.6–86.6)	(52.9–93.0)	(56.2–79.8)	(62.7–93.1)	(19.5–52.3)	(83.4–95.2)
Time flying (%) during:						
Daylight	57.7±9.7	58.7±10.8	56.5±7.0	60.1±6.2	77.8±4.1	29.2±5.1
	(45.7–73.4)	(40.3–75.5)	(42.3–65.3)	(50.1–70.7)	(71.0–84.7)	(21.1–37.7)
Darkness	25.7±10.2	24.1±11.6	33.1±7.5	22.1±8.6	59.1±8.7	10.5±3.8
	(13.4–44.4)	(7.0–47.1)	(20.2–43.8)	(6.9–37.3)	(47.7–80.5)	(4.8–16.6)
Total time on water (h):						
Daylight	6.0±1.5	6.5±1.5	7.6±1.2	5.9±1.0	2.9±0.5	11.2±0.7
	(3.6–7.9)	(3.9–9.0)	(5.8–9.6)	(4.2–7.5)	(2.0–3.8)	(10.0–12.3)
Darkness	7.2±1.0	6.0±1.3	4.1±0.7	7.1±0.9	4.4±0.9	7.1±0.3
	(5.7–8.5)	(3.6–7.9)	(3.3–5.4)	(5.8–9.1)	(2.2–5.7)	(6.5–7.5)
Total time in flight (h):						
Daylight	8.0±1.2	9.3±1.9	9.8±1.2	8.9±0.9	10.0±0.6	4.5±0.8
	(6.5–10.2)	(6.1–11.7)	(7.3–11.4)	(7.5–10.2)	(8.8–10.9)	(3.2–6.1)
Darkness	2.5±1.1	1.9±0.8	2.1±0.5	2.0±0.8	6.5±0.9	0.8±0.3
	(1.3–4.5)	(0.6–3.2)	(1.2–2.8)	(0.7–3.5)	(5.2–8.7)	(0.4–1.4)

Data for migration and the period of non-breeding residence are from Hedd et al. (2012). Values are means of individual bird means ±1 SD, with the range for individuals in parentheses.

### Oceanographic Characteristics

Prior to egg-laying, females concentrated their foraging (30% UDs) over the northern Patagonian Shelf and slope, and in the Argentine Basin, in areas that were deep (mean depth 2434 m during pre-breeding; 1647 m during pre-laying exodus), and relatively warm (mean SST 9.6°C and 9.9°C, respectively) with moderate productivity (mean chlorophyll *a* concentration 2.12 mg/m^3^ and 1.56 mg/m^3^, respectively; Table 3). Males at that time were centered mainly over shallow areas of the southern Patagonian Shelf (mean depth 346 m and 498), that were cooler and relatively less productive (mean SST 5.3°C and 7.5°C, and chlorophyll *a* concentration 1.24 mg/m^3^ and 0.89 mg/m^3^, respectively; Table 3). Both sexes used northern and southern areas during the pre-laying exodus (Fig. 4A), albeit in differing proportions, and habitat characteristics in more peripheral kernel regions were similar (Table 3). Throughout incubation, chick-rearing and the non-breeding period, when males and females shared foraging areas to a greater degree, habitat characteristics were similar (Table 3, Fig. 1E–J). The only notable difference was during incubation when productivity (chlorophyll *a* concentration) was relatively higher for females (Table 3).

**Table 3:** Bathymetric and oceanographic characteristics within the kernel density distribution of male and female Sooty Shearwaters during the pre-breeding, pre-laying exodus, incubation, chick-rearing and non-breeding periods.

Kernel	Water depth (m)		Sea Surface Temperature (°C)		Chlorophyll *a* concentration (mg/m^3^)	
	Males	Females	Males	Females	Males	Females
**Pre-breeding (October 2008)**						
0–30%	346±302	2434±2248	5.3±0.4	9.6±2.6	1.24±1.68	2.12±1.84
30–50%	633±488	2034±2056	5.4±0.5	8.5±2.5	1.37±3.08	1.54±1.66
50–90%	2106±2086	2752±2461	6.4±2.0	10.3±3.7	1.23±1.80	1.65±1.89
**Pre-laying exodus (November 2008)**						
0–30%	498±646	1647±1809	7.5±2.6	9.9±2.3	0.89±0.83	1.56±1.25
30–50%	1839±2297	2307±2414	9.0±3.0	10.1±3.3	1.77±2.88	1.34±1.95
50–90%	2156±2265	2636±2438	9.3±3.3	10.1±3.5	1.39±1.34	1.35±1.61
**Incubation (December 2008)**						
0–30%	553±603	361±381	7.0±0.6	8.0±1.0	0.54±0.23	1.73±3.95
30–50%	824±943	500±658	7.4±1.1	11.0±4.1	0.98±2.33	1.73±1.92
50–90%	1326±1558	1158±1529	7.8±1.7	11.9±3.4	1.49±2.41	1.62±1.46
**Chick-rearing (February 2008)**						
0–30%	537±613	318±277	8.9±1.1	9.4±0.9	0.91±2.10	1.16±2.38
30–50%	829±1099	732±657	8.2±1.4	9.1±1.2	0.71±1.54	0.84±1.77
50–90%	1650±1540	1485±1618	8.5±2.1	9.3±2.3	0.68±1.08	0.92±1.20
**Non-breeding residence (July 2008)**						
0–30%	115±42	133±48	13.0±1.0	13.2±1.0	0.25±0.07	0.34±0.17
30–50%	272±367	128±81	12.8±1.6	12.9±1.3	0.32±0.11	0.34±0.31
50–90%	1462±1473	1759±1769	15.2±3.3	15.1±3.1	0.34±0.59	0.32±0.53

Representative middle months were chosen for environmental extractions within the extended incubation, chick-rearing and non-breeding period.

## Discussion

Prior to egg-laying, when reproductive roles differed, sexually monomorphic male and female Sooty Shearwaters exhibited clear differences in marine distributions and behavior. From the time of initial return to the colony until egg-laying (i.e., including the pre-laying exodus), females travelled further from the colony to feed in deep waters that were warmer and relatively more productive than those used by males. Males, in contrast, made exclusively short foraging trips to nearby waters and attended the colony significantly more than females. Presumably due to their proximity to the colony, particularly during the pre-breeding period, males were also less active at sea during the day. The sexes essentially segregated at sea prior to egg-laying, with little overlap in core areas. Sex differences were reduced during incubation and absent during chick-rearing when trip durations, activity patterns and core foraging areas of males and females were very similar. Congruence between the sexes continued during the non-breeding period, when birds resided in the Northern Hemisphere. Segregation was therefore apparent only early in the breeding season, when there were clear differences in sex roles. Prior to egg-laying, males return regularly to the colony to defend the nest site or the mate, or to repair the burrow, while females are acquiring resources for egg formation, or subsequent breeding effort, by travelling to distant, yet relatively more productive waters.

Availability of small GLS devices has facilitated a number of recent studies of sexual segregation in other small to medium sized Procellariiformes. Similar to our findings for Sooty Shearwaters, female Manx Shearwaters *Puffinus puffinus*, also travelled further from the colony than males during the pre-laying exodus [8]. The authors speculated that areas used by females may have provided resources necessary for egg formation or simply that such areas were too far afield for males guarding the burrow. Again similar to colony attendance patterns described for Sooty Shearwaters, males of the Streaked *Calonectris leucomelas* and the Balearic Shearwater *Puffinus mauretanicus*, the former being strongly dimorphic, also spent more time at the colony, and had shorter duration foraging trips than females before egg-laying [33], [34]. Pre-laying exoduses of female Streaked Shearwaters were also significantly longer than those of males. The authors suggested that male Streaked Shearwaters needed to return to the colony more often to defend the nest site or mate, given that attendance patterns and foraging areas were similar during incubation [33] and this explanation seems likely to also apply to both Manx [8] and Sooty Shearwaters (this study). In two small, sexually monomorphic gadfly petrels, the Barau’s *Pterodroma baraui* and Chatham Petrels *P. axillaris*, there was also sexual segregation early in the season [14], [35]; however, in contrast to the situation for shearwaters, males of both *Pterodroma* species foraged further from the colony during the pre-laying exodus, and for Barau’s petrel males were more active at sea and foraged in more productive waters than females. No sex differences were observed during incubation or chick-rearing for Chatham Petrel [35] or during chick-rearing for Barau’s petrel [14], the latter leading the authors to postulate that sex-specific energetic requirements led to the unexpected behavioural differences early in the season. In order to regain condition after egg-laying, female Barau’s Petrels undertake an extended trip to sea; it was suggested that in order for males to prepare for this extended fast, they flew to more distant, productive waters during the exodus. Together, these recent studies suggest that sexual segregation in monomorphic Procellariiformes may be more widespread than previously thought; however, the variety of contributing processes indicate a need for further study.

With respect to seasonality, this and other recent tracking studies concur with conclusions from isotopic studies summarized by Phillips et al. [6], in that sexual segregation is more likely to occur during all or part of the pre-breeding or breeding period [14], [19], [33], [35], than during the non-breeding period. Sexual segregation apparent early in the breeding season in small (Barau’s and Chatham Petrels), medium (Streaked and Sooty Shearwaters) and large (Black-browed and Grey-headed albatrosses *Thalassarche melanophris* and *T. chrysostoma*) Procellariiformes had diminished by hatching, after which time parents presumably participated equally in raising their young [14], [33], [35], [36]. For Northern Gannets *Morus bassanus*, Wandering *Diomedea exulans*, Black-browed, Grey-headed and Light-mantled *Phoebetria palpebrata* albatrosses as well as Sooty Shearwaters in both the Atlantic (this study) and Pacific Oceans, there was no evidence of sexual segregation during the non-breeding period when energy demands are presumably reduced relative to the breeding season [19], [37]–[39].

There is a striking correspondence between the core foraging areas of pre-laying female Sooty Shearwaters (October-November) on the northern Patagonian Shelf, and the destination of both White-chinned Petrels from South Georgia during the pre-laying exodus (November; [29]) and of migrant Manx Shearwaters that spend the non-breeding period (October-March) in this region [8]. The Patagonian Shelf large marine ecosystem is one of the most productive regions in the southern hemisphere, supporting diverse and abundant zooplankton, squid and fish communities that are exploited by large-scale fisheries [40], [41], and resident and migrant marine top predators [8], [29], [42]–[44]. Surface chlorophyll-*a* concentrations over the northern Patagonian Shelf reach an annual maximum in October and November [45], which corresponds with the arrival of the Manx Shearwaters and the pre-laying exoduses of Sooty Shearwaters (this study) and White-chinned Petrels [29]. While the prey targeted by Sooty Shearwaters during this period is unknown, it seems likely that the area provides females with specific nutrients required for egg formation or that prey is sufficiently abundant in the region to offset travelling costs.

Although similar between the sexes, the distinct behavior of Sooty Shearwaters when rearing chicks in the Falkland Islands is notable. In contrast to the dual foraging strategy described for this species at breeding sites in New Zealand [46]–[48], birds in the Falklands almost exclusively performed short (∼1.4 day) foraging trips, and trip duration is clearly unimodal. Although reduced intra-specific competition could account for site differences (the Falklands population is tiny compared to that in New Zealand), an abundance of suitable foraging habitat relatively close to the colony seems a more likely explanation (see Phillips et al. [49]). The productive Patagonian Shelf has a complex oceanography that results in an apparent abundance of suitable foraging habitat close to the breeding colony. Similarly, Cory’s Shearwaters *Calonectris diomedea* breeding in the Canary Islands use a unimodal foraging strategy [50], but adopt a dual strategy at other sites [51]–[53] because the breeding colony lies close to highly productive areas over the NW African continental shelf. Supporting this contention, Paiva et al. [52] report a positive correlation between the percent of short trips performed by Cory’s Shearwaters at different breeding sites and the chlorophyll-*a* concentration of the surrounding waters.

As the mechanisms underlying seabird sexual segregation remain poorly understood [6], future studies that couple tracking and trophic investigations for monomorphic and sexually size dimorphic species will likely provide insights. Spatial segregation, in particular, can result in males and females being differentially exposed to anthropogenic threats (e.g., fisheries bycatch, hydrocarbon extraction activities, oil spills [3], [54], [55]) and to the biological consequences of ongoing environmental variation and change [3]. Year-round investigations of sexual segregation, facilitated by advances in tracking technology, can, therefore, provide valuable insights into seabird population dynamics, conservation and management [54].
